# Effects of informative and confirmatory feedback on brain activation during negative feedback processing

**DOI:** 10.3389/fnhum.2015.00378

**Published:** 2015-06-29

**Authors:** Yeon-kyoung Woo, Juyeon Song, Yi Jiang, Catherine Cho, Mimi Bong, Sung-il Kim

**Affiliations:** ^1^Department of Education, Brain and Motivation Research Institute (bMRI), Korea UniversitySeoul, South Korea; ^2^Department of Psychology, Rutgers UniversityNewark, NJ, USA

**Keywords:** negative feedback, informative feedback, confirmatory feedback, emotion regulation, dorsolateral prefrontal cortex, amygdala, functional MRI

## Abstract

The current study compared the effects of informative and confirmatory feedback on brain activation during negative feedback processing. For confirmatory feedback trials, participants were informed that they had failed the task, whereas informative feedback trials presented task relevant information along with the notification of their failure. Fourteen male undergraduates performed a series of spatial-perceptual tasks and received feedback while their brain activity was recorded. During confirmatory feedback trials, greater activations in the amygdala, dorsal anterior cingulate cortex, and the thalamus (including the habenular) were observed in response to incorrect responses. These results suggest that confirmatory feedback induces negative emotional reactions to failure. In contrast, informative feedback trials elicited greater activity in the dorsolateral prefrontal cortex (DLPFC) when participants experienced failure. Further psychophysiological interaction (PPI) analysis revealed a negative coupling between the DLPFC and the amygdala during informative feedback relative to confirmatory feedback trials. These findings suggest that providing task-relevant information could facilitate implicit down-regulation of negative emotions following failure.

## Introduction

Feedback plays an important role in facilitating individuals’ learning and optimizing their behavior. Negative feedback, in particular, helps individuals to monitor their performance and change their strategies in order to improve subsequent performance (Kluger and DeNisi, 1996; Holroyd and Coles, 2002). Literature on negative reward prediction error suggests such error signals provide useful information regarding how to modify one’s behavior, which could encourage individuals to regulate their goal-directed behaviors (Bischoff-Grethe et al., 2009; Kim, 2013).

Although previous research has found evidence for negative feedback supporting behavioral adjustment, negative feedback is also known to generate negative emotions such as frustration and anxiety that may lead to a decline in intrinsic motivation. In addition, actively regulating negative emotions induced by negative feedback demands the use of cognitive resources as demonstrated by the recruitment of executive cognitive control neural networks associated with emotion regulation (Goldin et al., 2008; Drabant et al., 2009). These findings suggest that the regulation of negative emotions is likely to compete with the cognitive resources required to successfully complete the task at hand, thereupon impairing performance in task-related activities (Pessoa et al., 2002b, 2005; Erthal et al., 2005; Ortner et al., 2013). As evidence of this, a study by Eysenck et al. (2007) found that emotional arousal impairs efficient processing of the goal-directed attentional system and increases attention towards affective aspects of the stimuli. Several neuroimaging studies have also revealed that negative emotions hinder performance in memory tasks, elevating amygdala activity in response to negative affect (Dolcos and McCarthy, 2006; MacNamara et al., 2011).

Findings from the above studies suggest negative emotional arousal can impede learning and disrupt performance in various tasks. Hence, minimizing negative emotions elicited from negative feedback is crucial for effectively processing feedback and achieving optimal levels of learning. Literature on feedback processing suggests certain forms of feedback may implicitly alter emotional responses by modifying the nature of information provided via feedback. Previous studies on negative feedback have distinguished several forms of feedback. For instance, confirmatory feedback refers to indications of the accuracy of responses, whereas informative feedback informs learners why their responses are correct or incorrect, providing with task-related information (Shute, 2008; Wang and Wu, 2008). Upon experiencing failure, receiving information only regarding the result of performance (i.e., confirmatory feedback) could elicit negative emotions by exerting attention towards the failure itself. In contrast, informative feedback with task-relevant information (i.e., the reason for failure) might serve to allocate attentional resources to the informative nature of the feedback in lieu of emotional reactions that arise upon receiving negative feedback. Previous experimental studies have suggested that providing informative feedback following failure could help individuals shift their attention away from the negative emotion generated by the negative feedback (Bangert-Drowns et al., 1991; Narciss and Huth, 2006; Hattie and Timperley, 2007). However, no neuroimaging research on feedback processing has directly compared the effect of feedback types on emotional responses upon receiving negative feedback.

Such automatic attentional control shares the same mechanism with implicit emotion regulation. While a large number of emotion regulation research has focused on instructing participants to voluntarily regulate their emotions (Ochsner and Gross, 2005; McRae et al., 2010), recent studies have reported that emotion can be modulated via the unconscious priming of subjects with words (Mauss et al., 2007; Williams et al., 2009), changing situational demands (Bargh and Morsella, 2008), or contextual manipulation (Mocaiber et al., 2011). Studies by Pessoa et al. (2002b, 2005) found decreases in amygdala activity in response to affective stimuli when individuals automatically exerted more attentional resources towards the task while engaging in a cognitive activity. Mocaiber et al. (2011) also found that contextual information could modulate brain responses towards emotional stimuli. In their study, amygdala and insula responses to unpleasant stimuli were automatically regulated when participants were told beforehand that the unpleasant pictures were taken from movie scenes rather than from real life. Moreover, Williams et al. (2009) directly compared the effectiveness of unconscious and conscious emotion regulation and found that implicitly priming subjects with reappraisal-related words facilitated the unconscious regulation of emotional experiences.

The present study aimed to investigate the effects of two types of negative feedback on brain activation. We used a block design to compare informative and confirmatory feedback, and manipulated the emotional intensity of the negative feedback by varying the facial expressions accompanying each feedback. In actual learning environments, people usually receive negative feedback accompanied by a variety of emotional expressions, such as angry or neutral faces. It has been well-established that angry faces elicit negative emotions and induce greater amygdala activity than do neutral faces (Pessoa et al., 2002a). Therefore, we introduced two emotional intensities for both informative and confirmatory negative feedback to identify whether the two forms of negative feedback could differentially affect neural responses depending on the level of emotional intensity. Thus, the present study varied two factors, feedback type and emotional intensity. Negative feedback consisted of two types (informative and confirmatory) and emotional intensity consisted of two levels (high and low).

We hypothesized that confirmatory feedback trials relative to informative feedback during failure would elicit greater activations in brain regions implicated in negative emotions, such as the amygdala. We also anticipated activations in the amygdala to vary depending on the emotional intensity of the confirmatory feedback. In contrast, we hypothesized that informative feedback consisting of task-relevant information would elicit greater neural activity in the dorsolateral prefrontal cortex (DLPFC) to reflect the cognitive control of negative emotions when participants experienced failure.

## Materials and Methods

### Participants

A total of 14 right-handed male undergraduates (mean age = 21.1 ± 2.2 years) were recruited online from three universities in Seoul, Korea. Students majoring in engineering science were recruited in order to minimize inter-individual variability among participants regarding spatial perceptual ability, a critical component of task performance in the present study. All participants were screened to ensure they were not using any medications and that they did not have a history of psychological or neurological disorders. Each participant signed a written informed consent form and all procedures were approved by the Korea University Institutional Review Board. After the experiment, participants received a compensation of 30,000 KRW (equivalent to approximately US$30) for their participation.

### Experimental Procedure

**Figure 1:**
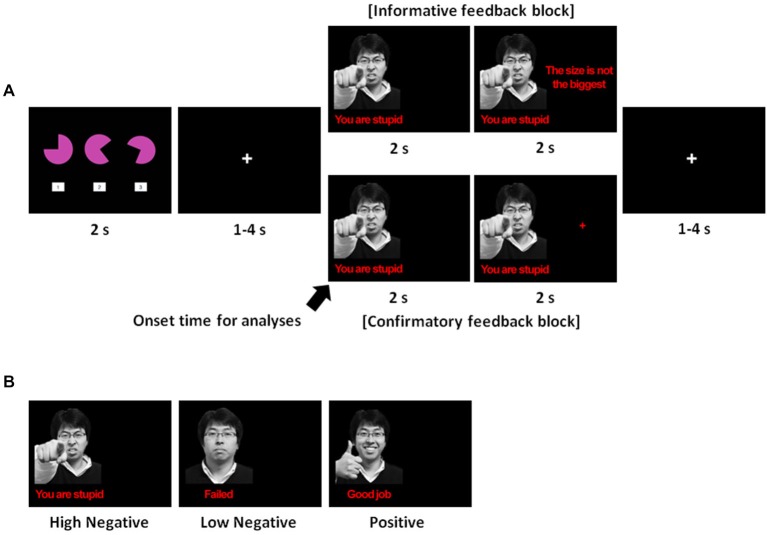
**(A)** Trial sequence. The spatial-perceptual task was presented for 2 s, during which participants had to select one of the three figures while satisfying all of the following criteria: (1) the selected figure must be largest in size; (2) the selected figure must have the largest cut-off angle; and (3) the participant must respond faster than the average response time based upon an undergraduate database. Following this, a fixation cross ranging from 1–4 s, with an average of 2 s appeared, and then feedback was displayed for 4 s. For the first 2 s, a facial expression with a verbal statement indicating whether the answer was correct or not was displayed on the left side of the screen. After the facial and verbal expression, additional information or a crosshair was presented for the next 2 s of the feedback phase depending on the type of feedback. Finally, a crosshair was displayed during a random inter-trial interval ranging from 1–4 s with an average of 2 s: **(B)** The three types of emotional feedback used in this study.

Upon arriving at the laboratory, all participants underwent a training session in which they received instructions about the spatial-perceptual task and performed a practice session on the computer. Instructions and experimental materials were presented and delivered in Korean. In each trial, participants were shown three geometric figures on a screen for 2 s and were asked to press one of three corresponding keyboard buttons to indicate their choice. For their choice to be marked correct, each response had to satisfy all of the following three criteria: (1) the figure had to be the largest in size; (2) the figure consisted of the largest cut-off angle; and (3) the subject had to respond faster than the average response time based on an undergraduate database (see Figure 1A). Participants were informed that there was one correct answer for each trial, and their responses would be considered a failure if their response was slower than the average response time. We developed a task consisting of multiple criteria because it enabled us to provide information about the cause of failure in each trial (i.e., the size, the angle of the figure, or the response time). Unbeknownst to the participants, however, feedback in each trial was predetermined in order to control for the number of positive and negative feedback presented to each participant. To ensure that the bogus feedback was credible to participants, the spatial-perceptual task was designed to be novel and ambiguous. During interviews after the experiment, all participants reported that they perceived the feedback to be credible and were based on their actual performance. All participants were fully debriefed after the experiment.

### Experimental Design

We used a mixed block/event-related design composed of three runs in total. Each run consisted of two blocks (informative feedback and confirmatory feedback) counterbalanced across participants. There were 20 trials in each block, resulting in a total of 120 trials. The presence of a blank screen (2 s) between every block informed the participants that the conditions were switching between informative and confirmatory feedback. Each trial lasted approximately 10 s on average. In each trial, three geometric figures were shown on the screen for 2 s. After selecting one of the three figures, feedback was presented for 4 s following a random fixation (1–4 s). After the feedback had been presented, there was a random inter-trial interval ranging from 1–4 s, with an average of 2 s. Figure 1A shows an example of a trial sequence.

The details of feedback manipulation were as follows. For the first 2 s of the feedback phase, emotional feedback was presented on the left side of the screen indicating whether or not the answer was correct (see Figure 1B). Emotional feedback consisted of the image of a face and a verbal statement. For an incorrect answer, participants were given two forms of negative feedback at different levels of emotional intensity. For a high-intensity negative feedback, the image of an angry face was accompanied by the derogatory statement, “You are stupid.” For a low-intensity negative feedback, the image of a neutral face was accompanied by the statement “Failed.” In contrast, for a correct answer, the image of a smiling face was presented alongside a complimentary statement, “Good job.”

Immediately after the emotional feedback, we differentiated the feedback type by manipulating whether or not task-relevant information was provided on the right side of the screen. For informative feedback, task-relevant information was presented on the right side of the screen. In these trials, when participants failed a trial, participants were informed about the criterion that they had missed (the size, the angle, or the response time). During trials in which the answer was marked correct, the phrase, “Please wait” was presented on the right side of the screen. For confirmatory feedback, a crosshair was displayed on the right side of the screen regardless of whether the answer was correct or not.

Although the primary focus of the current study was to examine negative feedback processing, it was necessary to include a number of correct trials in order for participants to believe the feedback was reliable. Accordingly, two-thirds of the trials provided negative feedback (of either high or low emotional intensity) and the remaining trials provided positive feedback. In addition, the frequency of negative feedback decreased and that of positive feedback increased as participants progressed through the trials in order to mimic learning trajectories in a real world setting. Each run consisting of 40 trials contained the different number of positive and negative feedback trials. In the first run, we provided 16 cases of high-intensity negative feedback, 14 of low-intensity negative feedback, and 10 of positive feedback. In the second run, we provided 12 instances of high-intensity negative feedback, 14 of low-intensity negative feedback, and 12 of positive feedback. In the third run, we provided 10 trials with high-intensity negative feedback, 12 with low-intensity negative feedback, and 18 with positive feedback. Furthermore, to prevent the habituation effect resulting from repetitive stimuli, particularly in regards to amygdala activation, we used the photos of nine different males during the feedback phase.

### fMRI Data Acquisition

The experiment was conducted at Ewha Womans University Medical Center. Images were acquired using a Philips 3T Intera Achieva MRI scanner (Philips Medical Systems, Andover, MA, USA). Functional images with blood oxygen level-dependent (BOLD) signal were acquired using a single-shot gradient echo planar imaging (EPI) sequence (TR = 2000 ms, TE = 30 ms, flip angle = 90°, FOV = 240 mm, ascending, 363 mm-thick slices, with no gap). Structural images were acquired after the first experimental run, and the high-resolution T1-weighted three-dimensional volume was acquired for anatomical localization (TR = 9.8 ms, TE = 4.6 ms, 160 slices, voxel size = 1 × 1 × 1 mm). Behavioral data were collected using E-PRIME v. 1.1. None of the participants exhibited head movement greater than 3 mm during the three runs.

### fMRI Data Analysis

Imaging data were analyzed using statistical parametric software (SPM 5, Wellcome Department of Imaging Neuroscience, London, UK) implemented in the Matlab (The MathWorks, Inc., Natick, MA, USA) environment. Imaging data for each participant were preprocessed and analyzed to delineate brain regions showing a BOLD signal change for each feedback type. Functional images were realigned to the first volume, corrected for the slice acquisition time, normalized to EPI templates implemented in SPM 5, and finally spatially smoothed using an 8-mm full width at half maximum (FWHM) isotropic Gaussian kernel.

After preprocessing, imaging data for each participant were analyzed using the general linear model (GLM) with the following regressors: six regressors for each type of feedback phase (i.e., the three types of emotional feedback in each informative and confirmatory block) and six regressors for the task phases following each type of feedback. In addition, participant response times during the task phases and the six head motion parameters from the realignment were also included as regressors of no interest in the statistical model.

Because we were particularly interested in neural activation corresponding to emotional regulation during negative feedback processing, individual contrast images were estimated by contrasting the beta values for the two levels of emotional intensity used in the delivery of the negative feedback. Thus, four contrast images were estimated. All individual contrast images were then collected to further examine the statistical significance of the evoked hemodynamic response in a second level random effect analysis. First, we conducted a whole-brain 2 × 2 (feedback type × emotional intensity) repeated measures ANOVA. The statistical criterion for the present study was a voxel-wise level of *p* < 0.05 false discovery rate (FDR) corrected for multiple comparisons, with a spatial extent cluster threshold (*k*) of five voxels.

Activations in *a priori* regions of interest (ROI) that failed to survive whole-brain correction were subjected to small-volume correction (SVC). ROI masks for SVC were created based on *a priori* anatomical structures rather than the activation observed from the present results. The mask for the amygdala was created using the automated anatomical labeling (AAL) atlas in the Wake Forest University PickAtlas (WFU PickAtlas) toolbox (Tzourio-Mazoyer et al., 2002; Maldjian et al., 2003). All coordinates were transformed from an Montreal Neurological Institute (MNI) template to Talairach coordinates, and the anatomical locations of significant neural activation foci were determined using the Talairach and Tournoux standard stereotaxic space (Talairach and Tournoux, 1988) and a standard brain atlas (Duvernoy, 1991). We also conducted ROI analyses by using the Marsbar toolbox (Brett et al., 2002) to infer and visualize the activation patterns of the regions identified in the ANOVA analyses.

PPI analysis (Friston et al., 1997) was conducted to assess functional connectivity of the ROI activations under the informative and confirmatory feedback conditions. We extracted the deconvolved time course from a 6 mm radius sphere centered on the DLPFC identified from the ANOVA analysis (see Table 1) as a seed region. The PPI analysis employed three regressors: one for the activation time course in a given volume of interest (the physiological variable), one for the experimental condition (informative feedback vs. confirmatory feedback; the psychological variable), and the last one for the regressor of interest which is the interaction between the time series of the seed region (the physiological variable) and the experimental condition (the psychological variable). PPI analysis was carried out for the ROI in each subject, and then entered into a random effects group analysis.

**Table 1:** **Brain regions from ANOVA analyses**.

Brain regions	BA	R/L	*N* of Voxels in cluster	Talairach coordinates			*z*-value
				*x*	*y*	*z*
**Main effect of feedback type**
Dorsolateral Prefrontal Cortex	46	L	9	−42	24	19	4.43*
Medial Frontal Gyrus	10	L	8	−14	45	12	4.47*
Dorsal ACC	32	L	8	−12	23	26	4.73*
Thalamus			60	−14	−31	5	4.85*
Parahippocampal Gyrus	36	R	32	34	−26	−17	4.82*
	36	L	9	−36	−34	−13	4.33*
Amygdala		R	27	30	−4	−20	3.15^†^
Superior Frontal Gyrus	8	L	17	−16	31	44	3.64
Postcentral Gyrus	3	R	13	57	−20	27	3.58
Cingulate Gyrus	24	R	16	10	−19	38	3.32
**Main effect of emotional intensity**							
Inferior Temporal Gyrus	37	R	13	51	−66	2	3.66

Note: *FDR whole-brain corrected; ^†^FDR small-volume corrected. *BA, Brodmann’s area; R/L, right or left hemisphere*.

Researchers have recently expressed concerns about non-independence in fMRI data analyses (Esterman et al., 2010), which refers to the repeated use of data from an initial statistical test in subsequent analyses. To circumvent this problem in our study, the leave-one-subject-out (LOSO) method was utilized for ROI and psychophysiologic interaction (PPI) analyses. To define independent clusters of an ROI, we carried out a group analysis that excluded the GLM data of a single participant. The beta weights for the excluded participant were extracted in the ROI defined by the group GLM from the remaining participants. The procedure was repeated for each participant.

## Results

### Behavioral Results

We tested for differences in reaction times depending on the type of feedback and emotional intensity of feedback. A 2 × 2 ANOVA revealed no significant differences between informative and confirmatory feedback, *F*_(1,13)_ = 2.79, *p* > 0.05, or between high-intensity and low-intensity negative feedback, *F*_(1,13)_ = 0.01, *p* > 0.05. There was no significant interaction between feedback type and emotional intensity in response times, *F*_(1,13)_ = 2.00, *p* > 0.05.

### fMRI Results

#### ANOVA Analyses

**Figure 2:**
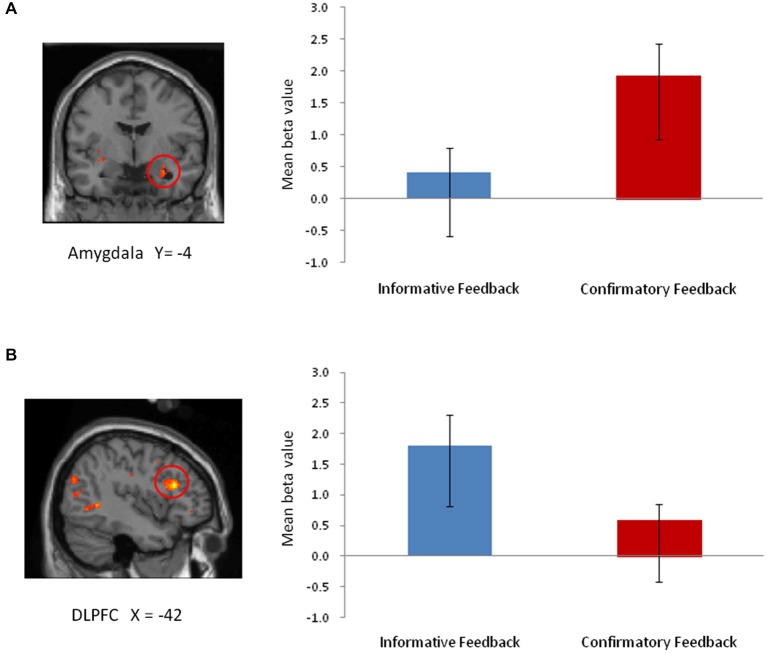
**The main effect of feedback type from 2 × 2 ANOVA. (A)** Amygdala (TAL coordinates: *x, y, z* = 30, −4, −20; 27 voxels). **(B)** DLPFC (TAL coordinates: *x, y, z* = −42, 24, 19; 9 voxels).

To identify regions that responded differentially to the two types of feedback and regions that were responsive to negative emotional intensity, we performed a whole-brain 2 × 2 (feedback type × emotional intensity) repeated measures ANOVA. We found feedback type significantly affected brain activation in two ROIs, including the amygdala (*p* < 0.05; FDR small-volume corrected) and the DLPFC (*p* < 0.05; FDR whole-brain corrected). As shown in Figure 2, ROI analyses revealed greater activity in the amygdala following confirmatory feedback compared to activity following informative feedback. The DLPFC, on the other hand, showed greater activity during informative feedback trials.

Previous neuroimaging studies have reported that increases in amygdala activation diminish over time with repeated exposures to stimuli (Breiter et al., 1996; Whalen et al., 1998). Therefore, to check for habituation effects in the amygdala in response to facial expression throughout the trials, we analyzed BOLD signals in the first five trials and compared them with the last five trials in each run. However, early vs. late trials resulted in no significant decrement of BOLD signal in the amygdala.

Besides the amygdala, confirmatory feedback trials also recruited the dorsal ACC (dACC) and thalamus (including the habenular, *p* < 0.05; FDR whole-brain corrected), regions previously implicated in negative emotions. Negative emotional intensity had a significant effect on inferior temporal gyrus activation (BA 37; 13 voxels; *p* < 0.001; uncorrected). However, no region emerged from the interaction between feedback type and emotional intensity, suggesting that similar regions were recruited for negative feedback regardless of emotional intensity of feedback. Table 1 summarizes the brain regions activated identified by the whole-brain ANOVA analysis.

We sought to identify brain regions associated with the effects of informative and confirmatory feedback on subsequent task performance by mapping neural activation during the task immediately following negative feedback. We thus analyzed task trials after receiving each type of feedback with a whole-brain 2 × 2 (feedback type × emotional intensity) repeated measures ANOVA. However, no significant brain activity in the fronto-parietal regions associated with spatial-perceptual processing was observed at the predefined statistical threshold.

#### Functional Connectivity Analysis

PPI analysis was conducted to access the brain regions that showed functional connectivity with the ROI activation. Specifically, we examined the functional connectivity patterns of the DLPFC during informative vs. confirmatory feedback trials (informative > confirmatory feedback). Because the DLPFC is a widely-established region responsible for cognitive control of emotions during affective processing, we were interested in whether increased activation of the DLPFC during informative feedback condition was accompanied by a decreased activation in the amygdala. A 6 mm radius sphere centered on the peak voxel of the DLPFC observed in the ANOVA analysis was identified as seed region for each participant when contrasting the informative feedback with confirmatory feedback (informative feedback with both high and low emotional intensity vs. confirmatory feedback with both high and low intensity). As shown in Table 2, PPI results revealed that during informative relative to confirmatory feedback conditions, a decreased activity in the amygdala (Talairach coordinates: *x*, *y*, *z* = −24, −8, −11, 23 voxels, *p* < 0.05, FDR small-volume corrected) was associated with an enhanced activation in the DLPFC. In addition, negative functional interactions between the DLPFC and both the bilateral thalamus (Talairach coordinates: *x*, *y*, *z* = −18, −15, 14, 21 voxels; 20, −17, 14, 13 voxels, *p* < 0.05, FDR small-volume corrected) were also detected from informative feedback vs. confirmatory feedback contrasts.

**Table 2:** **Brain regions from PPI analysis**.

Brain regions	R/L	*N* of Voxels in cluster	Talairach coordinates			*z*-value
			*x*	*y*	*z*
**Informative feedback > Confirmatory feedback**					
Amygdala	L	23	−24	−8	−11	4.00
Thalamus	L	21	−18	−15	14	4.19
	R	13	20	−17	14	3.99

*Note: All regions are significant at p < 0.05, FDR small-volume corrected with an extent threshold of 10 voxels. R/L, right or left hemisphere*.

## Discussion

The current study compared the differential effects of informative and confirmatory feedback on neural responses during negative feedback processing. The results revealed that negative feedback consisting of a facial expression and a verbal statement recruited different regions of the brain depending on whether the feedback was informative or confirmatory. Specifically, confirmatory feedback recruited a neural network associated with negative emotions, whereas informative feedback did not recruit these areas, but instead recruited the DLPFC. Previous evidence has implicated increased amygdala activity in response to unpleasant and negative emotions, such as fear and pain (Davis and Whalen, 2001; Hariri et al., 2003; Phelps and LeDoux, 2005) and also positive emotions (Sergerie et al., 2008; Mende-Siedlecki et al., 2013). In the present study, however, because we were only interested in outcomes with a negative valence, the recruitment of the amygdala in the current findings could be interpreted as processing negative affective information when receiving confirmatory feedback.

We also found increased activity in regions associated with negative emotional processing, such as the dACC and the thalamus (including the habenular) when individuals received confirmatory feedback. Activation of the dorsal region of the cingulate cortex has also been linked to the fear and threat responses (Milad et al., 2007; Wager et al., 2009; Etkin et al., 2011). In addition, the thalamus, especially the habenular, has been involved in the processing of aversive information and often demonstrates increased activity along with the amygdala when exposed to unpleasant stimuli (Lane et al., 1997; Hikosaka, 2010). The recruitment of neural circuitry involved in negative emotional processing, such as the amygdala, dACC, and thalamus, suggests that confirmatory feedback allocates attention towards the emotional component of a failure experience. These findings suggest that when participants fail, they are more likely to experience negative emotions and focus on the negative valence of confirmatory feedback than they would for informative feedback.

In contrast, we found that informative feedback trials did not recruit regions associated with negative emotion processing. During informative feedback trials relative to confirmatory feedback trials, we observed greater activity in the DLPFC, consistent with previous research implicating the prefrontal region in cognitive control of emotions during feedback processing (Jimura et al., 2004; Ridderinkhof et al., 2004). These findings are consistent with previous literature suggesting that the DLPFC is sensitive to the informative value of feedback, not to the valence of the feedback (Zanolie et al., 2008). Banich et al. (2000, 2009) also found that increased activity in the DLPFC has been linked with selecting and maintaining task-relevant information, and directing attention away from task-irrelevant information. Additionally, PPI analysis revealed a negative relationship between activations in the DLPFC and amygdala. Considering the role of the DLPFC in the down-regulation of the amygdala during emotion regulation (Pessoa et al., 2002b; Banks et al., 2007; Erk et al., 2007; Kanske et al., 2011), our results suggest the potential role of informative feedback in regulating negative emotions during negative feedback processing.

Together, these findings suggest that by providing additional task-relevant information, informative feedback promotes the implicit regulation of negative emotions as individuals anticipate necessary information required to improve performance. In our informative feedback blocks, task-relevant information was provided when participants made an error; as a result, individuals were able to expect receiving this information following failure. Attending towards task-relevant information could shift individuals’ attention away from the emotional feature of the negative feedback and direct attention towards potentially useful information for subsequent trials. In our paradigm, the use of a block design enabled participants to anticipate whether or not task-related information would be presented in upcoming trials, thus modulating emotional processing after receiving negative feedback. Collectively, these findings suggest feedback that includes task-relevant information could play a role in regulating negative affect by directing attention towards the task.

Findings from the present study contribute to the extant literature by demonstrating that individuals undergo different emotional processes during failure, depending on the type of feedback they receive. These findings suggest that without using deliberate emotion regulation strategies, negative emotions induced by negative feedback may be implicitly regulated by processing informative feedback. Therefore, we suggest that when it is necessary to provide negative feedback, the inclusion of task-relevant information explaining the causes of failure would be recommended for helping individuals shift their attention away from negative emotions and promote goal-directed behavior and maintain focus on the target task.

There are several limitations in the present study. First, feedback was predetermined regardless of the participants’ actual performance. We did this in order to maintain a similar level of performance and negative emotional intensity across all participants. If a suitable success rate could be manipulated for the task across individuals, participants’ actual performance could be measured following genuine feedback. Second, we mainly focused on investigating how different types of negative feedback influence individuals’ emotional and cognitive processes, so it remains necessary to explore positive feedback in a similar way. Third, we restricted our sample to male participants in our experiment in order to avoid potential gender differences in spatial ability because a large body of research has reported that males tend to score higher on mental rotation and spatial perception tests compared to females (e.g., Linn and Petersen, 1985). Therefore, future studies could test whether findings observed from the present study could be replicated with a sample of both genders. Lastly, careful interpretations of our results are necessary, due to our relatively small sample size.

## Conflict of Interest Statement

The authors declare that the research was conducted in the absence of any commercial or financial relationships that could be construed as a potential conflict of interest.
